# Genes regulating hormone stimulus and response to protein signaling revealed differential expression pattern during porcine oocyte in vitro maturation, confirmed by lipid concentration

**DOI:** 10.1007/s00418-020-01866-w

**Published:** 2020-03-18

**Authors:** Błażej Chermuła, Michal Jeseta, Patrycja Sujka-Kordowska, Aneta Konwerska, Maurycy Jankowski, Wiesława Kranc, Ievgeniia Kocherova, Piotr Celichowski, Paweł Antosik, Dorota Bukowska, Irena Milakovic, Marie Machatkova, Leszek Pawelczyk, Dariusz Iżycki, Maciej Zabel, Paul Mozdziak, Bartosz Kempisty, Hanna Piotrowska-Kempisty

**Affiliations:** 1 Division of Infertility and Reproductive Endocrinology, Department of Gynecology, Obstetrics and Gynecological Oncology, Poznan University of Medical Sciences, Poznan, Poland; 2 Department of Obstetrics and Gynecology, University Hospital and Masaryk University, Brno, Czech Republic; 3 Department of Histology and Embryology, Poznan University of Medical Sciences, 6 Święcickiego St., 60-781 Poznan, Poland; 4 Department of Anatomy, Poznan University of Medical Sciences, Poznan, Poland; 5 Department of Veterinary Surgery, Nicolaus Copernicus University in Torun, Toruń, Poland; 6 Department of Elementary and Preclinical Sciences, Nicolaus Copernicus University in Torun, Toruń, Poland; 7 Veterinary Research Institute, Brno, Czech Republic; 8 Chair of Biotechnology, Department of Cancer Immunology, Poznan University of Medical Sciences, Poznan, Poland; 9 Department of Histology and Embryology, Wroclaw Medical University, Wrocław, Poland; 10 Division of Anatomy and Histology, University of Zielona Gora, Zielona Gora, Poland; 11 Physiology Graduate Program, North Carolina State University, Raleigh, NC USA; 12 Department of Toxicology, Poznan University of Medical Sciences, Poznan, Poland

**Keywords:** Pig, Oocyte maturation, Microarray, Mitochondrial activity

## Abstract

Genes influencing oocyte maturation may be valuable for predicting their developmental potential, as well as discerning the mechanistic pathways regulating oocyte development. In the presented research microarray gene expression analysis of immature and in vitro matured porcine oocytes was performed. Two groups of oocytes were compared in the study: before (3 × *n* = 50) and after in vitro maturation (3 × *n* = 50). The selection of viable oocytes was performed using the brilliant cresyl blue (BCB) test. Furthermore, microarrays and RT-qPCR was used to analyze the transcriptome of the oocytes before and after IVM. The study focused on the genes undergoing differential expression in two gene-ontology groups: “Cellular response to hormone stimulus” and “Cellular response to unfolded protein”, which contain genes that may directly or indirectly be involved in signal transduction during oocyte maturation. Examination of all the genes of interest showed a lower level of their expression after IVM. From the total number of genes in these gene ontologies ten of the highest change in expression were identified: *FOS*,* ID2*,* BTG2*,* CYR61*,* ESR1*,* AR*,* TACR3*,* CCND2*,* EGR2* and *TGFBR3*. The successful maturation of the oocytes was additionally confirmed with the use of lipid droplet assay. The genes were briefly described and related to the literature sources, to investigate their potential roles in the process of oocyte maturation. The results of the study may serve as a basic molecular reference for further research aimed at improving the methods of oocyte in vitro maturation, which plays an important role in the procedures of assisted reproduction.

## Introduction

Determination and characterization of the expression of genes which regulate the hormone stimulus and response to protein signaling in mature oocytes is valuable for predicting their developmental potential. It has been already proven that evolutional capacity of oocytes is acquired in the long stages of folliculogenesis (Rienzi et al. 2011; Rybska et al. 2018a). In this process, the main role is played by the bi-directional communication between the oocyte and cumulus cells (CCs) (Celichowski et al. 2018). Corona radiata cells, directly contacting the oocyte, supply it with molecular regulating factors and necessary nutrients. The main goal of all these complex development processes is ensuring the nuclear and cytoplasmic cellular maturity of the oocyte (Kahraman et al. 2018), which is achieved through gap junctions between the cells of the cumulus-oocyte complexes (COCs). Results of previous research illustrated that the cooperation of somatic cells surrounding the oocyte is important for the functioning of the signaling and metabolic pathways that enable the oocyte to reach metaphase II (Chamier-Gliszczyńska et al. 2018).

The proper development of ovaries and ovarian follicles requires appropriate gene expression. During oocyte maturation, many genes, such as the TGF-β receptor-associated genes, are involved in the activation of signaling pathways. TGF-βs are cytokines from the family of intercellular signaling factors that are mainly responsible for regulating proliferation, differentiation and embryonic development (Mauviel 2005). TGF-β receptor is the main cellular receptor responsible for morphogenesis and oocyte maturation. Firstly, the ligand binds to the TGF- β receptor on the cell surface, which initiates a series of molecular signals, inducing serine-threonine kinase activity.

The meiosis of human oocytes begins during embryonic development and stops in late prophase. When women reach sexual maturity, the appearance of a luteinizing hormone (LH) wave directly affects the granulosa cells, which surround and are in bi-directional communication with the oocyte, releasing the ovum from its arrested state and preparing it for further fertilization (Kranc et al. 2018; Rybska et al. 2018b). The inhibition of preovulatory vesicle meiotic progression is dependent, inter alia, on the cyclic guanosine monophosphate nucleotide (cGMP) and series of proteins that diffuse from the granulosa cells through the gap junctions. Recent works suggest that LH surge generates protein signals that are transferred to the oocyte in the same way as cGMP (Shuhaibar et al. 2015). Taking the above into account, it seems interesting to examine the impact of the expression of oocyte genes involved in the signaling associated with the meiosis resumption process.

Identifying and characterizing genes regulating hormone stimulus and response to protein signaling may prove to be important for assessing their expression during immature oocyte at prophase I stage maturation in the assisted reproduction techniques (ART). Thanks to oocyte in vitro maturation (IVM) application in humans ART procedures, the use of controlled hyper-stimulation could be eliminated. It would undoubtedly have a positive clinical effect in patients suffering from PCOS (polycystic ovary syndrome). PCOS patients are highly sensitive to gonadotropin stimulation, and it may consequently lead to the appearance of ovarian hyperstimulation syndrome (OHSS) (Vuong et al. 2020). Thus, a thorough understanding of the processes regulating porcine oocyte maturation in in vitro conditions can contribute to increasing the effectiveness of ART techniques in animals breeding as well as in assisted reproduction techniques used in humans.

Implementation of the microarray expression method for routine genetic diagnosis enabled the identification of new cell functions involved in the development of the ovarian follicle (Dias et al. 2018; Kranc et al. 2018; Nawrocki et al. 2018) and is a helpful tool for gene transcript analysis in cells and tissues (Celichowski et al. 2018; Chamier-Gliszczyńska et al. 2018; Chronowska 2014). The objective of the study was to evaluate the expression of genes expressed in porcine oocytes to define genes undergoing differential expression in two analyzed ontology groups: “Cellular response to hormone stimulus” and “Cellular response to unfolded protein”, using microarray gene expression analysis of immature and in vitro matured porcine oocytes. The resulting analysis of gene of interest expression patterns may provide us with new insight into the mechanisms of oocyte growth and maturation in in vitro culture.

## Materials and methods

Large portions of the Materials and Methods section were based on other works from the same cycle of studies, employing similar experimental procedures (Budna et al. 2017; Celichowski et al. 2018).

### Experimental design

Porcine oocytes were either classified as (1) BCB positive (+) for molecular analysis or (2) BCB positive (+) oocytes that were matured in vitro (IVM).

### Animals

A total of 45 Landrace gilts, with an average age of 155 days (range 140–170 days) and an average weight was 100 kg (95–120 kg) were maintained under identical and standard management practices. All experiments were approved by the Poznan University of Medical Sciences Bioethical Committee (Resolution No. 32/2012, approved on 1/6/2012).

### Porcine ovaries and cumulus-oocyte complexes (COCs)

Ovaries and reproductive pathways were recovered during slaughter and transported to the laboratory within 40 min, at 37 °C in 0.9% NaCl. Subsequently, ovaries were placed in a 5% fetal bovine serum solution (FBS, Sigma-Aldrich Co., St. Louis, MO) dissolved in PBS. Single large follicles (> 5 mm) were then punctured using a 5 ml syringe with a 20G needle in a sterile Petri dish to recover the COCs, which were placed in a Petri dish, COCs washed three times with PBS supplemented with 50 μg/ml, gentamycin 36 μg/ml of pyruvate and 0.5 mg/ml bovine serum albumin (BSA; Sigma-Aldrich, St. Louis, MO, USA). COCs were selected under an inverted microscope (Zeiss, Axiovert 35, Lübeck, Germany), counted and morphologically evaluated using the system presented by Jackowska et al. (2009). COCs of Grade I, characterized by homogeneous ooplasm and compact CCs, were the only ones considered in subsequent stages of the experiment. 300 first degree oocytes (3 ×* n* = 50 immature groups, 3 × *n* = 50 IVM groups) were collected.

### Hematoxylin and eosin ovaries staining

Ovaries collected from 5 porcine specimen were mixed in Bouin's solution for 48 h, dehydrated, cleared and embedded in paraffin. Sections of ovaries (3–4 μm) were cut using a semi-automatic rotary microtome (Leica RM 2145, Leica Microsystems, Nussloch, Germany) and stained employing a routine hematoxylin and eosin (H and E) procedure. Histological sections were evaluated using a light microscope, with selected pictures taken with a high-resolution scanning technique and Olympus BX61VS microscope scanner (Olympus, Tokyo, Japan).

### Selection of oocytes by BCB staining

**Fig. 1:**
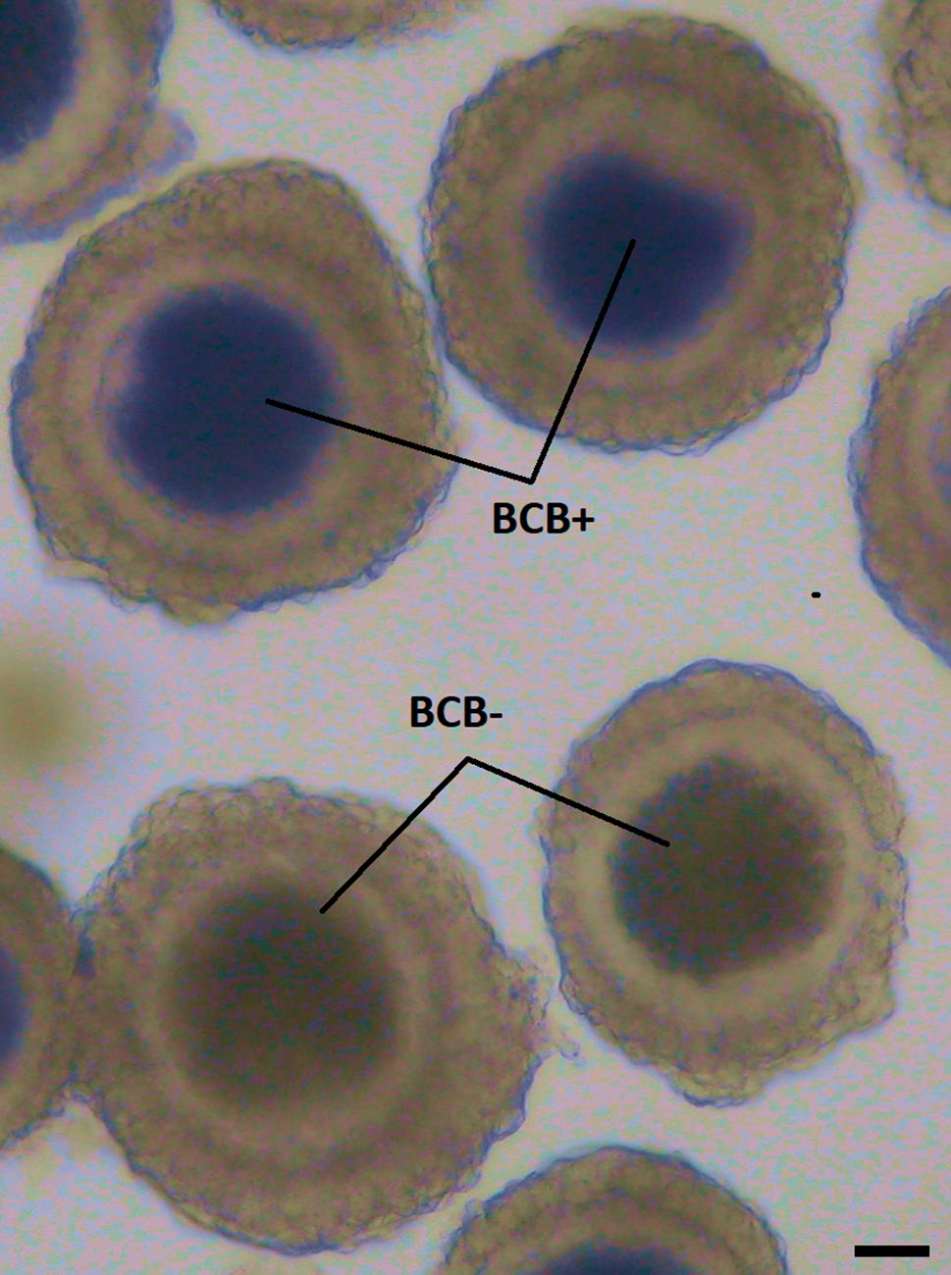
Briliant cresyl blue (BCB) staining of porcine oocytes. Representative picture of BCB+ and BCB− oocytes after their staining by BCB. During the washing procedure, oocytes were examined under an inverted microscope and classified as blue (BCB+) or colorless (BCB−). Only BCB+ oocytes were used for subsequent molecular analysis (immature group) or IVM. The contrast of the Figure was globally adjusted to better visualize the difference between blue and colorless oocytes

The BCB staining assay was performed to select oocytes for subsequent tests (Kempisty et al. 2011). This test bases on the activity of the glucose-6-phosphate, described to be lower in growth oocytes and higher in those undergoing development. This enzyme has the ability to convert the BCB staining from blue to colorless, hence serving as a good indicator of oocyte’s maturational competence, with BCB+ oocytes proven to be larger and more competent for maturation (Wu et al. 2007). Oocytes were rinsed twice with modified Dulbecco PBS (DPBS) (Sigma-Aldrich, St. Louis, MO, USA) supplemented with 50 μg/ml streptomycin, 50 IU/ml penicillin (Sigma-Aldrich, St. Louis, MO, USA), 0, 4% BSA [w/v], 0.34 mM pyruvate and 5.5 mM glucose (DPBSm). The oocytes were treated with 13 μM BCB (Sigma-Aldrich, St. Louis, MO, USA) diluted in DPBSm at 38.5 °C and 5% CO 2 for 90 min. Oocytes were transferred to DPBSm and washed twice. During the washing procedure, the oocytes were examined under an inverted microscope and classified as either blue (BCB+) or colorless (BCB-). Only BCB+ oocytes were used for subsequent molecular analysis (immature group) or IVM, followed by a second BCB test and molecular analysis (IVM group). An example of the BCB staining assay, showing samples of both BCB+ and BCB− oocytes can be found on Fig. 1.

### Denudation of porcine oocytes

Immature oocytes have dense layers of cumulus cells. Thus, COCs were first incubated with bovine testicular hyaluronidase (Sigma-Aldrich, St. Louis, MO, USA) for 2 min at 38 °C to separate cumulus and granulosa cells. The cells were then removed by vortexing in 1% sodium citrate buffer, followed by mechanical displacement by means of a small diameter glass micropipette. Both in the case of the immature oocytes purification and those after in vitro maturation process, denudation was carried out until corona radiata cells were completely removed from oocyte zona pellucida.

### Culture of cumuls-oocyte complexes

COCs were cultured in 4-well Nunclon™ plates in 500 μl of standard IVM porcine culture medium TCM-199 (tissue culture medium) with Earle salts and l-glutamine (Gibco BRL Life Technologies, Grand Island, NY, USA), supplemented with 0.1 mg/ml sodium pyruvate (SigmaAldrich, St. Louis, MO, USA), 2.2 mg/ml sodium bicarbonate (Nacalai Tesque, Inc., Kyoto, Japan) 10 mg/ml BSA, (SigmaAldrich, St. Louis, MO, USA), 0.1 mg/ml cysteine (Sigma-Aldrich, St. Louis, MO, USA), 10% filtered porcine follicular fluid (v/v) and gonadotropin supplements at final concentrations of 2.5 IU/ml of eCG (Intervet, Whitby, ON, Canada) and 2.5 IU/ml of hCG (Ayerst Laboratories, Inc., Philadelphia, PA, USA). All wells were flooded with a layer of mineral oil and maintained for 44 h at 38 °C in a 5% CO_2_ atmosphere. After maturation, for the maturity assessment the BCB staining test was repeated, with only BCB+ oocytes used for further study.

### RNA isolation

Oocytes were analyzed before (3xn = 50) and after in vitro maturation (3xn = 50). Each group was combined into three independent samples representing separate experimental groups. Total RNA was extracted from the samples with RNeasy MinElute Kit Cleanup (Qiagen, Hilden, Germany) and TRI Reagent® (Sigma, St Louis, MO, USA), according to the manufacturer protocols and literature data (Chomczynski and Sacchi 1987). Total mRNA was measured using the optical density at 260 nm, and the purity of RNA from the absorption ratio 260/280 nm (above 1.8) (NanoDrop spectrophotometer, Thermo Scientific, ALAB, Poland). The RNA integrity was determined with the use of Bioanalyzer 2100 (Agilent Technologies, Inc., Santa Clara, CA, USA). Obtained RNA integrity (RIN) was ranging from 8.5 to 10 with an average of 9.2 (Agilent Technologies, Inc., Santa Clara, CA, USA). Each sample was separated and secured for further analysis. 100 ng of the obtained RNA was used for microarray assays. The remaining RNA was used for the RT-qPCR analysis.

### Microarray expression analysis and statistics

The Affymetrix procedure was described and used in our previous works regarding porcine oviductal cells (Kranc et al. 2018; Stefańska et al. 2018) and porcine oocytes (Borys-Wójcik et al. 2018; Budna et al. 2018). All experiments were carried out in triplicate. Total RNA (100 ng) from each sample was subjected to two rounds of sense cDNA amplification (Ambion® WT Expression Kit). cDNA was used for biotin marking and fragmentation by labeling and hybridization to the Affymetrix GeneChip® WT (Affymetrix, Santa Clara, CA, USA). Biotin-labeled cDNA fragments (5.5 μg) were hybridized to the Affymetrix® Porcine Gene 1.1 ST Array Strip (48 °C/20 h). The microarrays were then washed and stained according to the technical protocol presented by Affymetrix GeneAtlas Fluidics Station. The array strips were scanned using the GeneAtlas system imaging station. The initial analysis of the scanned chips was carried out using the Affymetrix GeneAtlas™ operational software. The quality of gene expression data was checked in accordance with the quality control criteria provided by the software.

All analyses were carried out using the BioConductor software, accordingly to the statistical R programming language. Robust Multiarray Averaging (RMA) algorithm implemented in the “affy” BioConductor package was used for normalization and summation of raw data and background correction. The biological annotation was taken from the BioConductor “oligo” package, in which the object, data frame and commentary were combined with a normalized data set, which led to the full gene data table. Statistical significance of the analyzed genes was carried out with the use of moderated t-statistics from the empirical Bayes’ method. Obtained p-value was corrected for multiple comparisons using the Benjamini and Hochberg false discovery index. The choice of significantly altered gene expression was based on a p-value less than 0.05 and a greater fold expression change than |2|.

Functional grouping of gene annotations was performed using DAVID (Database for Annotation, Visualization and Integrated Discovery). Gene symbols for up- and down-regulated genes from each compared group were transferred into DAVID with the use of BioConductor “RDAVIDWebService” package. For further analysis, we chose extended GO terms, which had at least 5 genes and a *p* value (Benjamini) lower than 0.05. Improved GO conditions were subjected to a hierarchical clustering algorithm and were presented as heat maps.

The relationship between genes belonging to selected GO terms were analyzed with the GOplot package. The GO Plot package calculated the z-score: number of up-regulated genes minus the number of down-regulated genes divided by the square root of the number. This information allowed us to estimate the course of change of each gene ontology term.

Interactions between genes with different expression/proteins belonging to the selected ontological groups were tested by using STRING10 software (Search Tool for the Retrieval of Interacting Genes). The list of gene names was used as a query to predict interactions. Search criteria based on the coexistence of genes/proteins in scientific texts (text exploration), co-expression and experimentally observed interactions. That analysis generated a gene/protein interaction network in which the intensity edge reflects the strength of the interaction results. In addition to predicting interaction, STRING also allowed us to perform functional enrichment of GO terms based on previously detected gene sets.

Functional interaction between the genes belonging to selected GO BP terms was analyzed by the REACTOME FIViz application for the Cytoscape 3.6.0 software. The ReactomeFIViz aims to find paths and patterns of networks related to cancer and other types of diseases. This application allows finding the paths stored in the Reactome database, enabling route enrichment analysis for the gene set, visualization of hit paths using manually arranged path diagrams directly in Cytoscape and testing functional compounds between genes in the pathways of interest. The application can also access the Reactome Functional Interaction (FI) network, which spans over 60% of human proteins with high reliability.

### Quantitative analysis of polymerase chain reaction in real time (RT-qPCR) analysis

For RT-qPCR, total RNA isolated earlier from oocytes groups before and/or after IVM was used. RNA samples were resuspended in 20 μl of RNase-free water and stored in a – 80 °C freezer. RNA samples were treated with DNase I and reverse transcribed (RT) into cDNA. RT-qPCR was performed in a real-time Light Cycler PCR detection system (Roche Diagnostics GmbH, Mannheim, Germany) using SYBRR Green I as a detection dye, with target cDNA quantified using a relative quantification method. The relative abundance of analyzed transcripts in each sample was normalized to internal standards (PBGD, B-ACTIN, 18S rRNA). For amplification, 2 μl of diluted cDNA was added to 18 μl PCR QuantiTectR SYBRR Green PCR (Master Mix Qiagen GmbH, Hilden, Germany) and primers (Table 1). To provide negative control for subsequent PCR, one RNA sample from each preparation was analyzed without an RT reaction.

**Table 1:** Sequences of primers used for the RT-qPCR analysis

Gene	Gene ID	Primer sequence (5′–3′)	Product size (bp)
*AR*	397582	GGCAAAAGCAACGAAGAGAC	188
		CGACTCGGATAGGCTGCTAC	
*BTG2*	100048932	AACTCTCCCTGCTCCTCTCC	203
		TGAGGATCCAGCCATAGTCC	
*CCND2*	397162	CGTCCAAGCTCAAAGAGACC	169
		CGAAGAATGTGCTCGATGAA	
*CYR61*	100153791	CTAGATGCTGCTCGGGTTTC	246
		AGCTCCAAAATGAAGCAGGA	
*EGR1*	100520726	AGGTCACCATGGAAGGTCTG	152
		TCCAAAATCCATGCAAATCA	
*EGR2*	100038004	ACCCAGAAGGCATCATCAAC	232
		GAGGGGTCCTGGTAGAGGTC	
*EIF2AK3*	100513348	CTCCAGGACAGCTGCCTTAC	192
		CATTCTGGGCTCTTCTTTGC	
*ESR1*	397435	AGCACCCTGAAGTCTCTGGA	160
		TGTGCCTGAAGTGAGACAGG	
*FOS*	100144486	TCGGTAAGCGTCTGAGAGGT	209
		AAAGCTCTTGCCATGCAGTT	
*HSPA4*	100524853	CAGGATTTGCCCTATCCAGA	180
		CGGTTCCTCATTTTCCTCAA	
*ID1*	100522839	CCTCTGTTTTCCCATCTGGA	211
		ATGCCTCCCCTTTCTCATCT	
*ID2*	654298	CCCAAGGAGGACAAGTCAAA	180
		TCCCCATGGTGGGAATAGTA	
*IGFBP7*	100302573	TGACACCCCCTAAGGACATC	173
		CGTCTGAATGGCCAGGTTAT	
*IHH*	397174	CTCCACTGCCCTCTCAGAAC	182
		AGCTCGCAGCTGTGTCACTA	
*INSR*	396755	GTGCCCGACCATCTGTAAGT	245
		CCTGCCTCCTTGAGTTCTTG	
*KLF10*	100153665	AGGTTGTGAACGGAGGTTTG	160
		TAGCTTCTTGGCCGACAGAT	
*MMP14*	397471	TCCAGAACTACACCCCCAAG	185
		GCTGTCACCATGGAAACCTT	
*PHIP*	100155644	TAAAAGCTGCAGGCATGTTG	167
		CAGTTGGAACAAGTCGCTCA	
*PLD1*	100519446	CCTCCGTAATGGATGGAAAA	161
		CGCTGTTGAAACCCATACCT	
*SERPINH1*	396773	CGTGGGTGTCACTATGATGC	192
		TTCCCCATCCAGATCTTCAG	
*TACR3*	100521983	TCATTTATGCGCTTCACAGC	156
		GTCTGGGTTTCAAGGGATCA	
*TGFBR3*	397512	TTTGTTTTAGCTGGGGGTTG	177
		TGGCCACAGGGATTTTTAAG	
*TXNIP*	733688	TCGAAGTGATGGATCTAGTGGA	151
		TCACCTTCACAGAACCCTTTT	
*UBE2B*	100513527	TGGCATCTCCACTATGAGCA	164
		ACCTGCCTTGTTCAACCAAC	
*VCP*	397524	ACCCTCCAAGGGAGTGCTAT	223
		GGCAATTGAATCCAGCTCAT	

### Lipid droplet examination

**Fig. 2:**
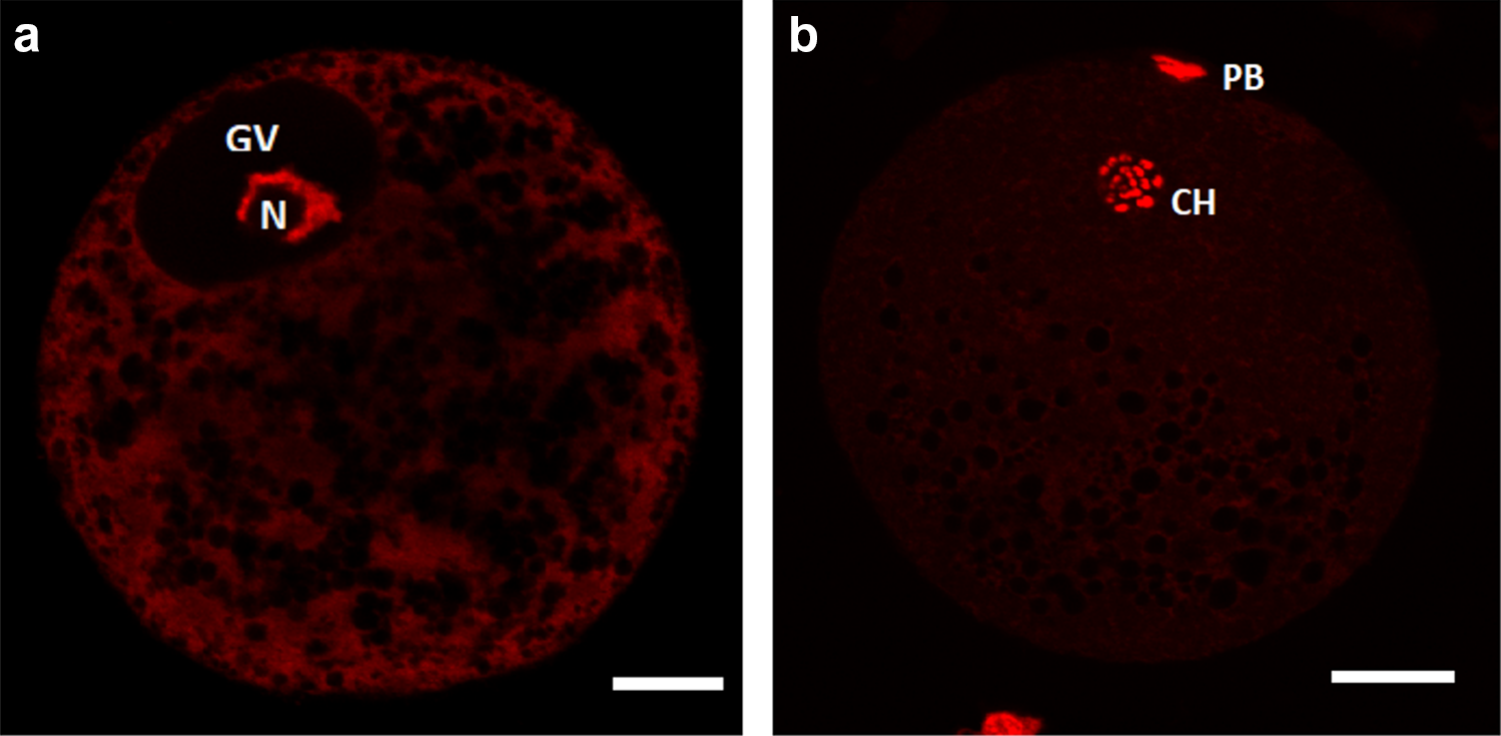
Sample images taken during fluorescence microscopy confirmation of oocyte maturity. **a** Immature oocyte after isolation at germinal vesicle stage (GV), **b** mature oocyte after 44 h of cultivation at metaphase of second meiotic division (MII); *N* nucleolus, *PB* polar body, *CH* condensed chromosomes. Scale bar represents 30 µm

The procedure of this step was based on sources found in the available literature (Bradley et al. 2016). Oocytes in germinal vesicle stage and mature oocytes, with the stages confirmed with the use of fluorescence microscopy methods (Fig. 2), were denuded of cumulus cells manually by narrow glass pipette in IVM culture medium with 0.1% (w/v) hyaluronidase (Sigma Aldrich). 10 oocytes from each group were fixed in 3.7% paraformaldehyde solution for 60 min at room temperature. Subsequently the oocytes were washed in PBS and permeabilized with 1% Triton X-100 for 1 h. The lipid droplets were stained in PBS supplemented with 0.4% BSA and 1 µM Nile red dye (Invitrogen, Carlsbad, CA, USA) at room temperature for 10 min. The oocytes were washed three times in PBS, with a single equatorial slice of each oocyte mounted on slide, avoiding oocyte compression, using Vectashield medium (Vector Lab, Burlingame, CA) containing 1 μM of DNA dye (TO-PRO-3, Invitrogen; Carlsbad, CA, USA) for either GV or MII stage control. The slides were stored below 0 °C until the oocyte examination.

The oocytes were examined using a laser scanning confocal microscope (Leica TCS SP2 AOBS; Leica, Heidelberg, Germany) equipped with Ar and HeNe lasers. The 488 nm excitation band and 570–667 nm detector were used for lipid droplets visualization and 633 nm excitation band and 635–713 nm detector for detection of chromatin. The 40× Leica HCX PL APO CS objective, pinhole, offsets, gain and AOBS were adapted. These parameters were kept throughout the whole experiment. The oocytes were scanned in equatorial optical sections, and microphotographs were saved and processed using the NIS-Elements AR 3.00 software (Laboratory Imaging).

The data were analysed with Fisher’s least significant difference (LSD) test using ANOVA SPSS version 11.5 for Windows (SPSS, Inc., Chicago, IL, USA). Differences at *p* < 0.05 were considered statistically significant. Differences at *p* < 0.05 were considered statistically significant.

## Results

**Fig. 3:**
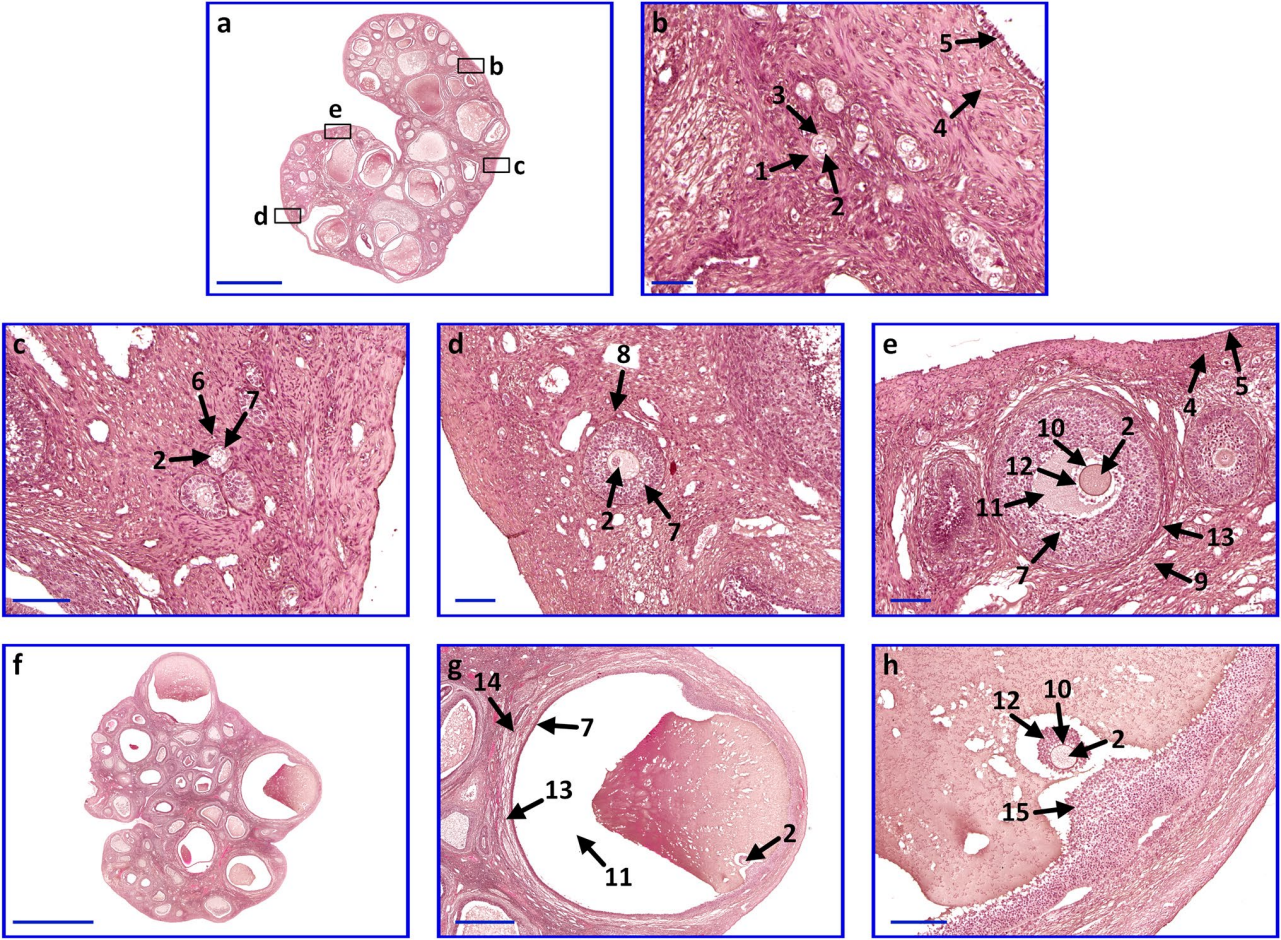
Microphotograph representing ovaries with follicles in different stages of development. **a**–**h** ovaries and follicles. Arrows: 1, primordial follicle; 2, oocyte; 3, follicular cells; 4, tunica albuginea; 5, germinal epithelium; 6, unilaminar primary follicle; 7, granulosa cells; 8, multilaminar primary follicle; 9, secondary follicle; 10, zona pellucida; 11, antrum; 12, corona radiate; 13, theca interna and theca externa; 14, Graafian follicle; 15, cumulus oophorus. Scale bar sizes: **a**, **f **5000 µm; **g** 1000 µm; **h **200 µm; **c**–**e** 100 µm; **b **50 µm

Hematoxylin and eosin staining allowed to determine the histological structure of collected ovaries. Each of the samples revealed proper morphology. Follicles in different stages of development: primordial, primary unilaminar, primary multilaminar, secondary and Graafian follicle were noted (Fig. 3).

**Fig. 4:**
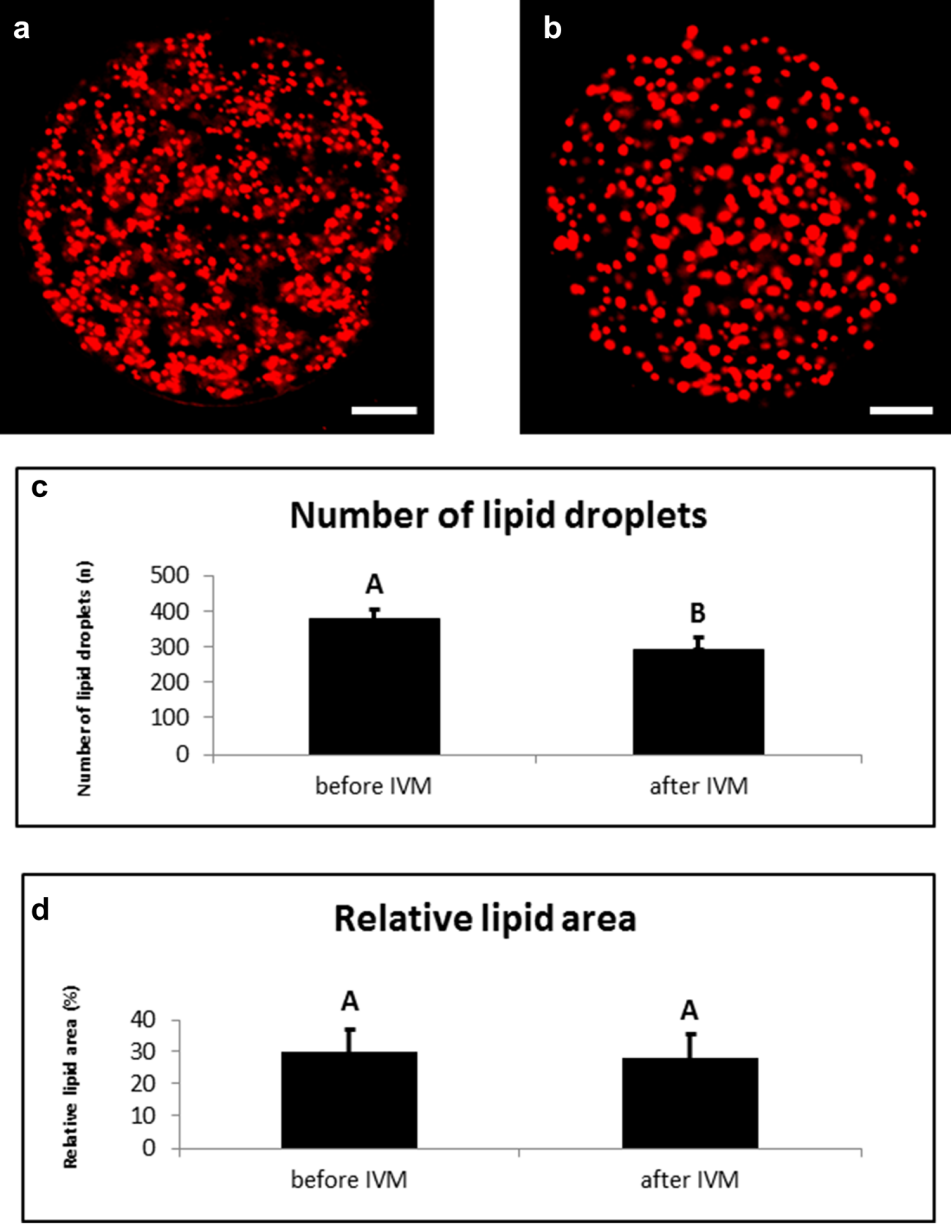
Contents of lipid droplets during IVM. Representative images of porcine oocytes before (**a**) and after (**b**) in vitro maturation. Oocytes were stained by Nile red (red colour—lipid droplets). Scale bar represents 20 µm. (**c**) Number of lipid droplets in porcine oocytes before (0 h) and after maturation (44 h). The oocytes were examined by confocal microscopy after either collection or maturation in standard conditions. (**d**) Relative lipid area in an optical section of scanned porcine oocytes before (0 h) and after maturation (44 h). The oocytes were examined by confocal microscopy after either collection or maturation in standard conditions. Values with different superscripts are significantly different (**a**, **b**, *p* < 0.05)

The results of lipid droplet examination indicate that the mean values of the total lipid composition for immature oocytes were higher than those in the mature oocytes. The total number of lipid droplets of immature porcine oocytes was significantly higher in comparison to oocytes with expanded cumulus (Fig. 4). During the process of maturation, the total number of lipid droplets decreased in oocytes in comparison to the germinal vesicle stage.

However, the results suggest that the areas covered with lipid droplets are similar in the immature and mature oocytes, indicating that the porcine oocytes contain a lower number of lipid droplets of larger surface after maturation (Fig. 4).

Whole transcriptome profiling by Affymetrix microarray allows the analysis of gene expression changes in freshly isolated oocytes, before in vitro procedure (“before IVM”), compared to after in vitro maturation (“after IVM”). Using Affymetrix® Porcine Gene 1.1 ST Array we have examined expression of 12,258 porcine transcripts. Genes with a fold change higher than |2| and with a corrected p-value lower than 0.05 were considered as differentially expressed, and there were 419 different transcripts revealed via the analysis. Subsequently, the genes were used for the identification of significantly enriched GO BP terms.

**Fig. 5:**
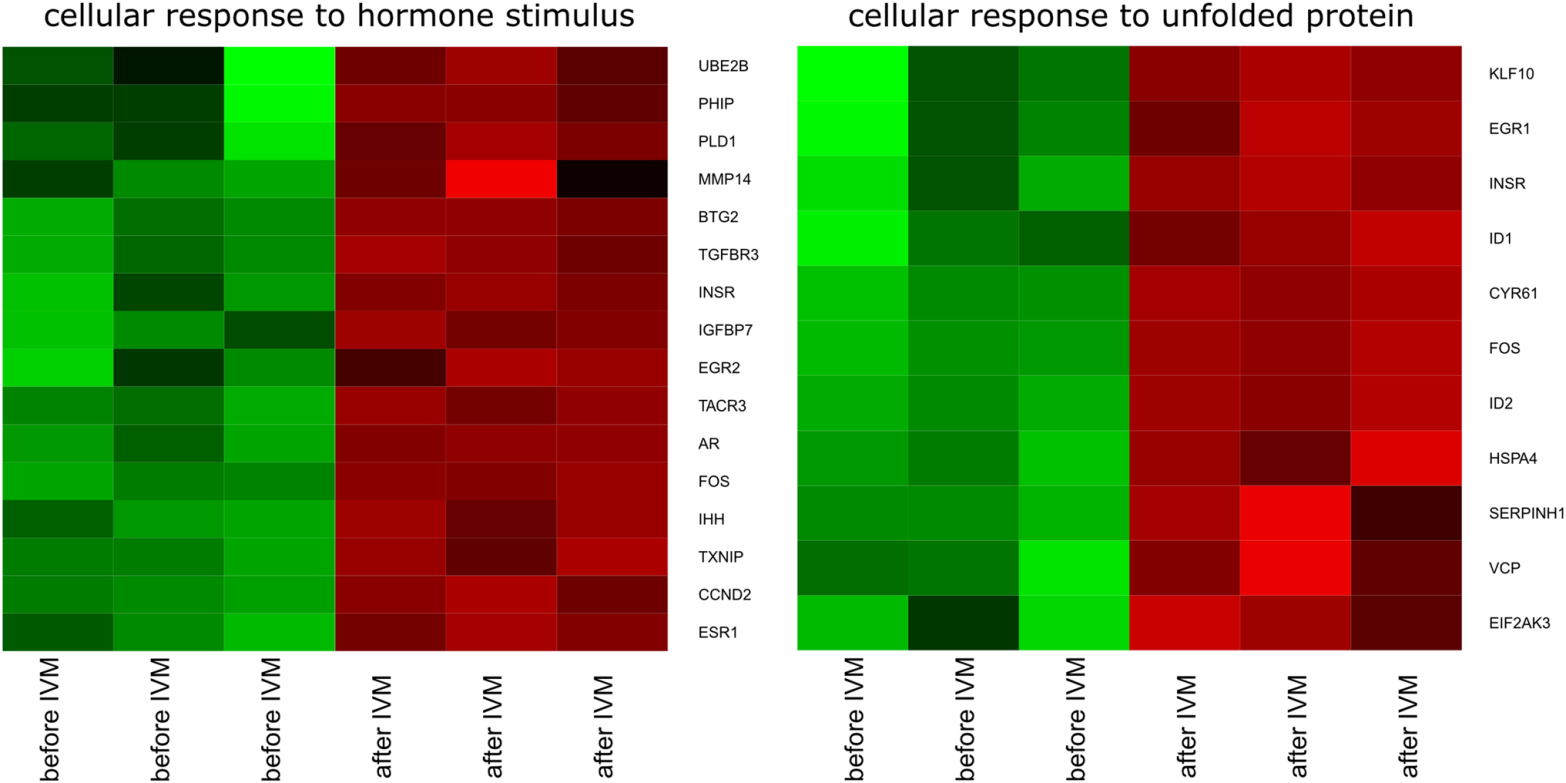
Heat map representations of differentially expressed genes belonging to the “cellular response to hormone stimulus” and “cellular response to unfolded protein” GO BP terms. Arbitrary signal intensity acquired from microarray analysis is represented by colours (green, higher; red, lower expression). Log2 signal intensity values for any single gene were resized to Row Z-Score scale (from − 2, the lowest expression to + 2, the highest expression for a single gene)

DAVID (Database for Annotation, Visualization and Integrated Discovery) software was used for extraction of the genes belonging to “cellular response to hormone stimulus” and “cellular response to unfolded protein” gene ontology Biological Process terms (GO BP). 25 genes from these GO BP terms were significantly represented in down-regulated gene sets (Fig. 5).

The set of the differentially expressed genes belonging to “cellular response to hormone stimulus” and “cellular response to unfolded protein” GO BP terms with their official gene symbols, fold changes in expression and corrected *p* values was also shown in Table 2.

**Table 2:** Gene symbols, fold changes in expression and corrected p values of studied genes

Gene	Fold change	Adj. *p *value	Entrez gene ID
*AR*	0.105986	0.000138	397582
*BTG2*	0.074386	9.55E−05	100048932
*CCND2*	0.121809	0.000179	397162
*CYR61*	0.080657	7.54E-05	100153791
*EGR1*	0.376128	0.005477	100520726
*EGR2*	0.165504	0.00795	100038004
*EIF2AK3*	0.41889	0.008422	100513348
*ESR1*	0.08163	0.000522	397435
*FOS*	0.052794	4.74E−05	100144486
*HSPA4*	0.441182	0.002321	100524853
*ID1*	0.335473	0.003974	100522839
*ID2*	0.06298	4.74E−05	654298
*IGFBP7*	0.40376	0.002496	100302573
*IHH*	0.304996	0.000551	397174
*INSR*	0.316016	0.001913	396755
*KLF10*	0.405439	0.006845	100153665
*MMP14*	0.488721	0.03806	397471
*PHIP*	0.385682	0.021116	100155644
*PLD1*	0.468342	0.011045	100519446
*SERPINH1*	0.467321	0.006338	396773
*TACR3*	0.11506	0.000148	100521983
*TGFBR3*	0.196522	0.000406	397512
*TXNIP*	0.355539	0.000781	733688
*UBE2B*	0.38278	0.041105	100513527
*VCP*	0.435612	0.007402	397524

**Fig. 6:**
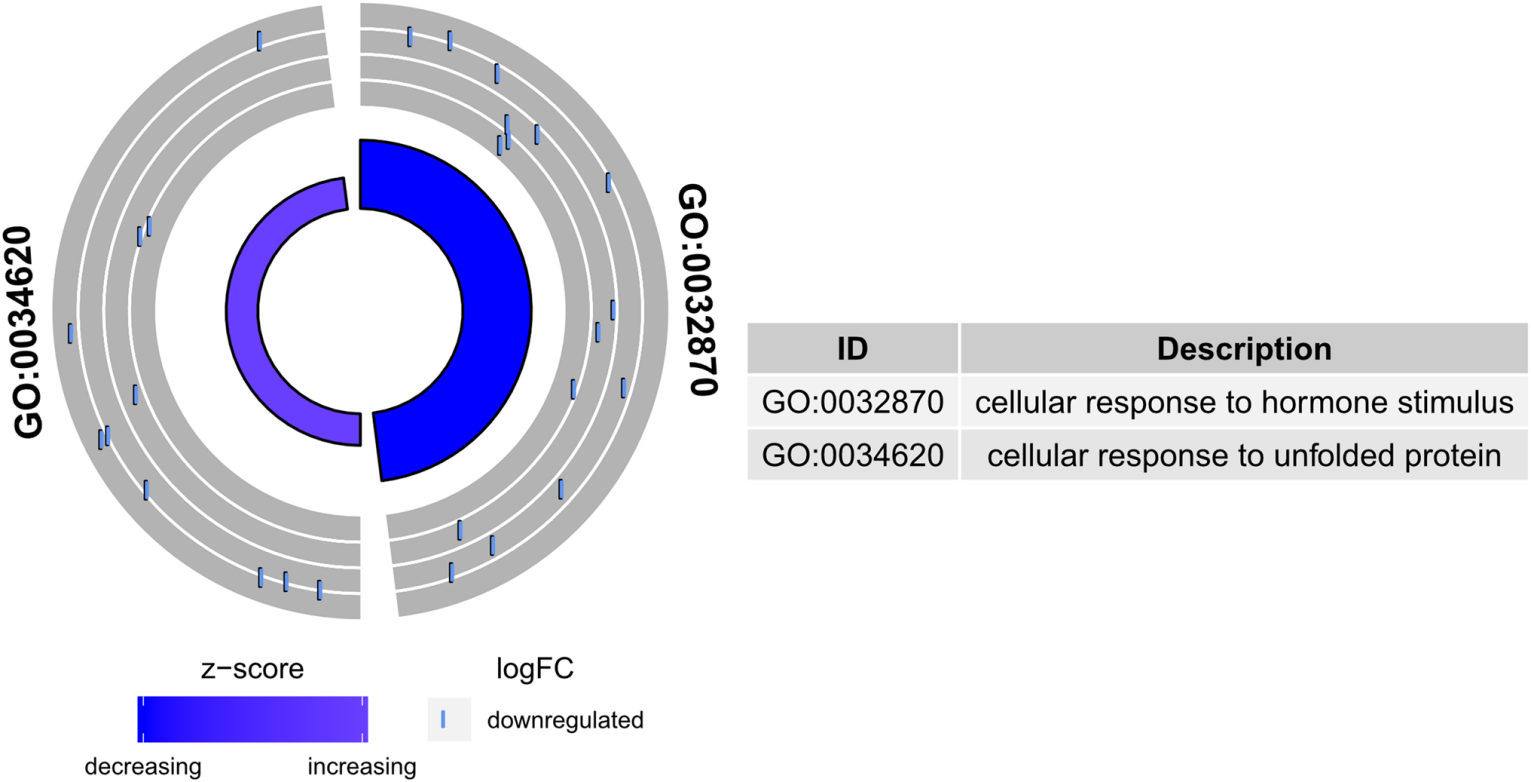
The circle plot showing the differently expressed genes and z-score “cellular response to hormone stimulus” and “cellular response to unfolded protein”. The outer circle shows a scatter plot for each term of the fold change of the assigned genes. Purple circles display up-regulation and blue ones down-regulation. The inner circle shows the z-score of each GO BP term. The width of each bar corresponds to the number of genes within GO BP term and the color corresponds to the z-score

The enrichment of each GO BP term was calculated as a z-score and shown on the circle diagram (Fig. 6).

**Fig. 7:**
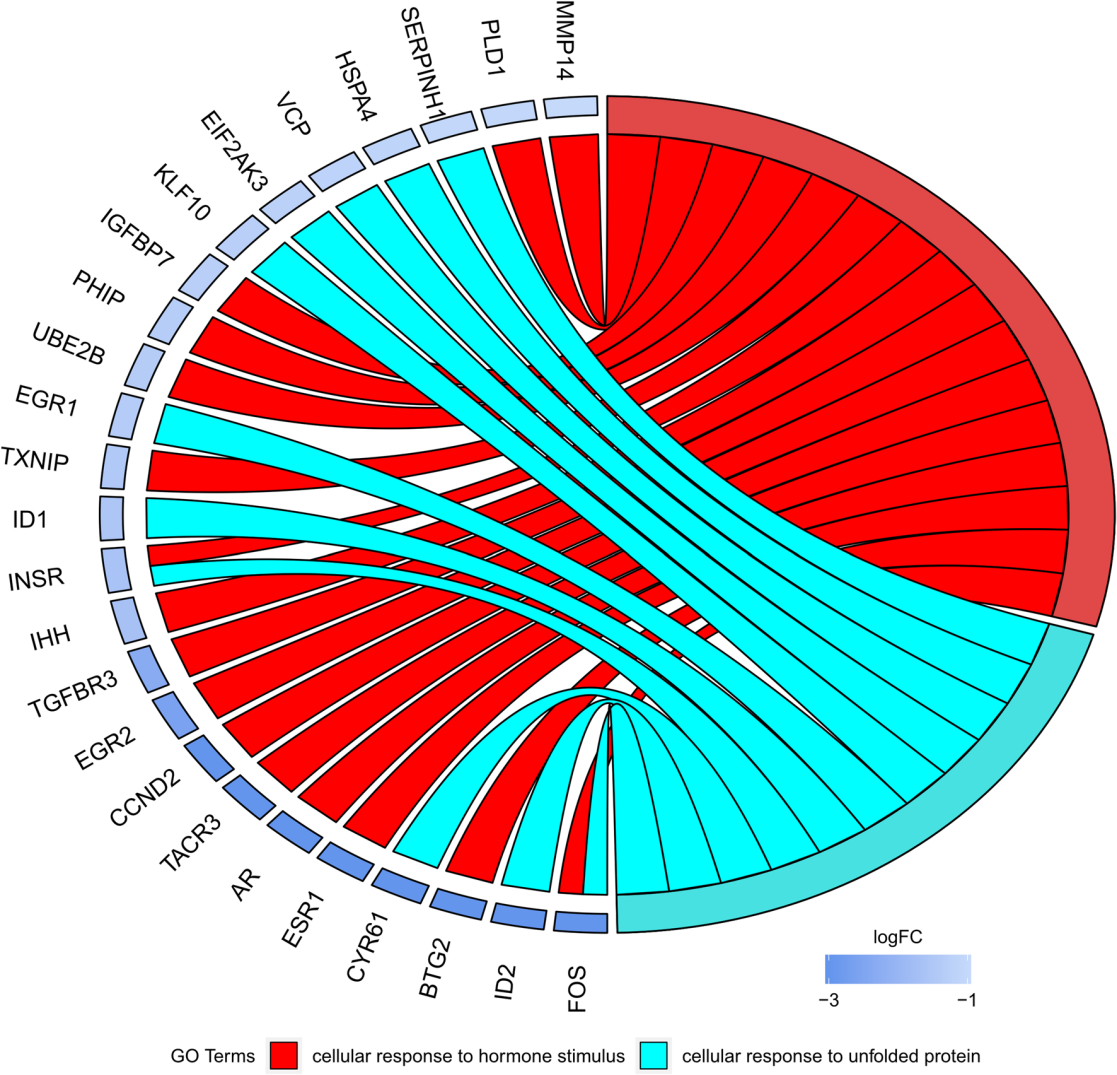
The representation of the mutual relationship between differently expressed genes that belong to the “cellular response to hormone stimulus” and “cellular response to unfolded protein”. The ribbons indicate which gene belongs to which categories. The middle circle represents logarithm from fold change (LogFC) after IVM. The genes were sorted by logFC from most to least changed gene

**Fig. 8:**
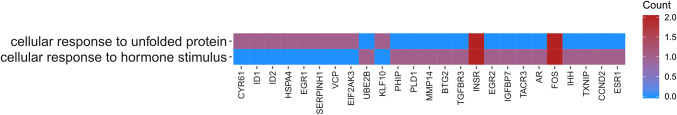
Heatmap showing the gene occurrence between differently expressed genes that belongs to the “cellular response to hormone stimulus” and “cellular response to unfolded protein”. The red color is associated with gene occurrence in the GO Term. The intensity of the color is corresponding to the amount of GO BP terms that each gene belongs to

Moreover, in the Gene Ontology database, the genes that form one GO group can also belong to other different GO term categories. For this reason, we explore the gene intersections between selected GO BP terms. The relation between those GO BP terms was presented as a circle plot (Fig. 7) as well as a heatmap (Fig. 8). Most of the presented genes belong to one of the GOs of interest. Only two of the genes: INSR and FOS, are members of both of the analysed gene ontologies.

**Fig. 9:**
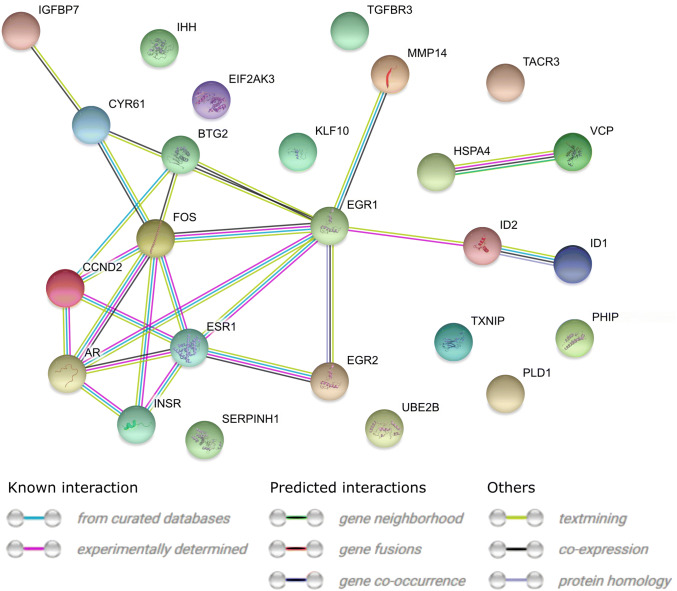
STRING-generated interaction network between genes that belongs to the “cellular response to hormone stimulus” and “cellular response to unfolded protein”. The intensity of the edges reflects the strength of the interaction score

**Fig. 10:**
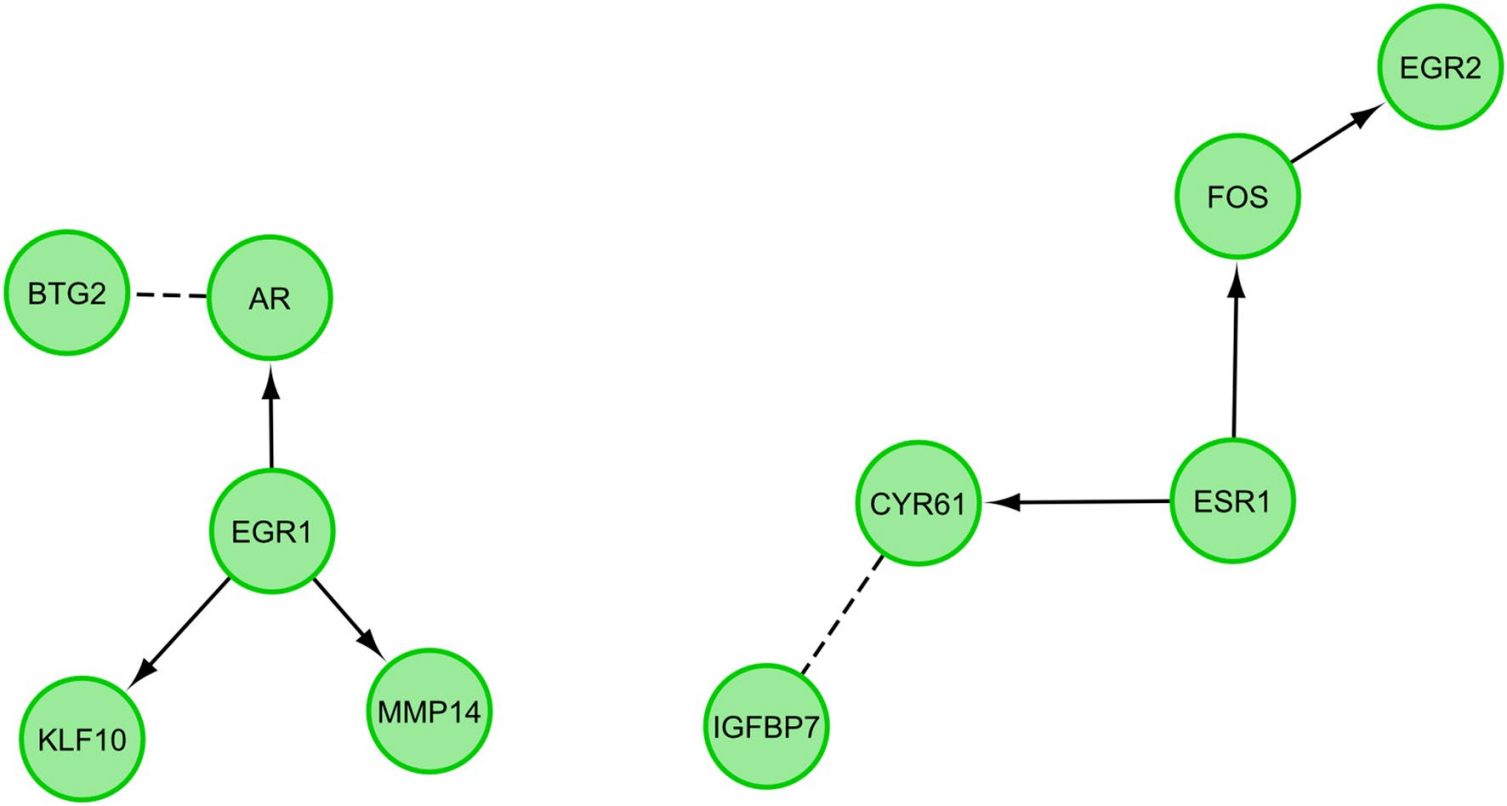
Functional interaction (FI) between genes that belongs to the “cellular response to hormone stimulus” and “cellular response to unfolded protein”. In the following figure “→” stands for activating/catalyzing inputs, and “---” for predicted FIs

STRING-generated interaction network was created between differentially expressed genes belonging to the studied ontology terms. The intensity of the edges reflects the strength of the interaction score (Fig. 9). The functional interactions between chosen genes were investigated using the REACTOME FIViz app to the Cytoscape 3.6.0 software. The results were shown in Fig. 10.

**Fig. 11:**
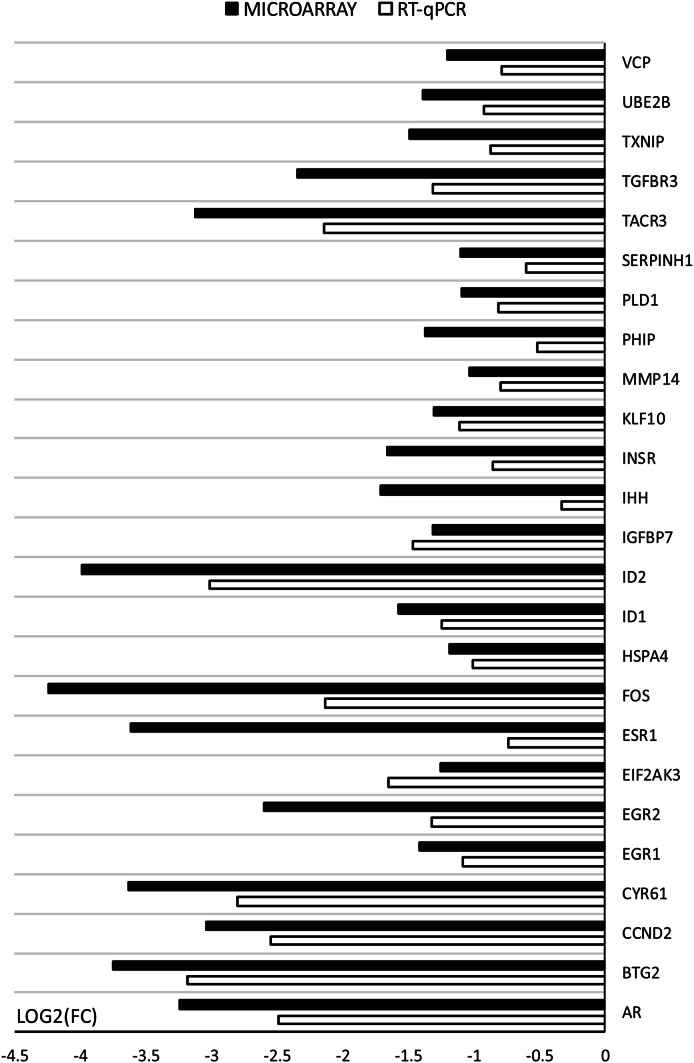
Comparison of gene expression analysis of oocytes before IVM (3xn = 50) and after IVM (3xn = 50) using microarray assay and RT-qPCR. RT-qPCR analysis was normalized to the expression of three housekeeping genes (PBGD, β-actin, 18S rRNA). All of the differences in expression were statistically significant at: *p* < 0.05

Microarray results were validated using RT-qPCR (Fig. 11), and the differential regulation of genes was confirmed via both methodologies. However, the scale of changes of some of the genes varies between the methods, which may be explained through their different sensitivity and specificity. All of presented genes showed the same pattern of expression as in the microarray analysis.

## Discussion

Molecular interactions with the surrounding somatic cells are a key element during oocyte maturation in the course of folliculogenesis process (Chermuła et al. 2019). There are already many reports of these dependencies, but they are still not well understood. The possibilities offered by the analysis of gene expression using the microarray technique allowed to define and determine the processes occurring during the development of ovarian follicles (Bryja et al. 2018; Chermuła et al. 2018). Microarray technology provides a comprehensive analysis of tissue transcriptome and is a tool that creates the possibility of understanding molecular bases and relationships between the genes driving physiological and pathophysiological processes. Transcriptomic analysis was carried out on two groups of oocytes: before and after the in vitro maturation process. Additionally, to confirm their metabolic maturity, lipid profile was determined in pre-mature oocytes, as well as those subjected to in vitro maturation. The results of the analysis provided a possible confirmation of oocyte maturity (as illustrated by lipid droplets reduction after oocyte maturation) with a method independent of molecular maturation. Furthermore, histological staining of sample ovaries serving as a source of the oocytes analyzed in this study was performed. The resulting figures aim to confirm the proper physiology of the ovarian tissues, as well as visualize the location of the maturing oocyte in the growing follicle in in vivo conditions.

The analysis of genes belonging to the “Cellular response to hormone stimulus” and “Cellular response to unfolded protein” ontology groups was the focus of the current study. These two gene groups may be directly or indirectly involved in signal transduction during oocyte maturation in both in vivo and in vitro (IVM) conditions. Examination of all the 25 genes of interest allowed to observe a reduced level of expression after IVM in all of them. In total, from 25 differentially expressed genes, ten: *FOS*, *ID2*, *BTG2*, *CYR61*, *ESR1*, *AR*, *TACR3*, *CCND2*, *EGR2* and *TGFBR3*, were identified as being significantly downregulated after in vitro maturation.

The first of the analyzed genes, with the largest change in expression after the maturation process, is *FOS* (Fos proto-oncogene) gene. The protein encoded by this gene, together with other proteins, forms the AP-1 transcription factor subunit (Borys et al. 2018).

One of the most important functions of this gene is cell differentiation, transformation and proliferation (Krishna et al. 2018). It has been suggested that the expression of this gene drastically increases in ovulatory follicles after administration of human chorionic gonadotropin (hCG). It is one of the key ovulatory genes required to increase the level of prostaglandins (PG) during ovulation in human granulosa cells (Choi et al. 2018). Earlier reports indicate the presence of *FOS* transcripts in germinal vesicles (GV) and metaphase II (MII) oocytes (Regassa et al. 2011). Decreased level of *FOS* gene expression after oocyte maturation observed in this study confirms the results presented by Blaha et al. (2015). These results prove that the amount of *FOS* mRNA rapidly and dramatically decreases during the first hour of the oocyte-cumulus complex (COC) culture. It was noted that *FOS* mRNA concentration gradually decreased throughout the entire culture period. These combined results may prove that in MII stage oocytes, the demand for FOS transcripts is at a much lower level than in the immature COCs. Considering the above, the most important function of this gene is the regulation of hormone stimulation and protein signaling activation during oocyte maturation process in growing ovarian follicles.

The second most downregulated gene, present only in “Cellular response to unfolded protein” ontology group, is *ID2* (inhibitor of DNA binding 2). *ID2* encodes a protein with a negative effect on cellular differentiation (Massari and Murre 2000). In ovarian follicles, the highest level of *ID2* protein is observed in granulosa cells, with expression of this gene stimulated by *GDF9* (growth differentiation factor 9) and *BMP15* (bone morphogenetic protein 15). In addition, FSH promotes *ID2* expression in porcine granulosa cells (Blaha et al. 2015). In poultry, *ID2* mRNA is involved in control and differentiation of follicles, as well as differentiation of granulosa cells. Hogg et al. (2010) suggested that ID proteins play one of the main roles in growth regulation and differentiation of ovarian steroidogenic cells. Expression of ID family proteins is, in various ways, regulated by members of the TGFβ family through intracellular SMAD signaling pathways. Lower *ID2* gene expression, observed in the “after IVM” group of oocytes, may result from the fact that expression of ID proteins in growing in vivo ovarian follicles is subject to paracrine stimulation through activation of activin and/or BMPs through the SMAD signaling pathway (Zhang et al. 2018).

The gene of the third most changed expression, a member of “cellular response to hormone stimulus” ontology group, is *BTG2* (anti-proliferation factor 2). The protein encoded by this gene has antiproliferative properties. BTG2 proteins were involved in the regulation of the G1/S stage transition during the cell cycle. Reduced expression of this gene is observed in human tumors, possibly due to weak differentiation of tumor cells (Hu et al. 2013). It has also been proven that *BTG2* can direct cells towards the apoptotic process (Kranc et al. 2018). The ovarian inflow of LH induces the expression of BTG family proteins that play an important role in the transfer of follicular granulosa cells into luteal phase through regulation of kinetics of their cell cycle. The low level of gene expression observed in mature oocytes is due to the fact that oocytes do not have apoptotic properties and do not differentiate into cells with different traits during the course of obtaining cellular maturity for fertilization (Kranc et al. 2018). *CYR61* (cysteine-rich angiogenic inducer 61) encodes a proliferation and adhesion mediating protein expressed on the cell surface. Reduced expression of *CYR61* is noted in tumor tissues (Chien et al. 2004). *CYR61* is a potential molecular mediator of the CL (corpus luteum) angiogenesis. Steroidogenic and endothelial cells of the corpus luteum are a source of *CYR61* expression (Chermuła et al. 2019). Reduction of this gene’s expression results in partial oocyte separation from the surrounding granulosa cells causing the loss of bi-directional communication with future CL.

The six other analyzed genes, such as *BTG2,* only belong to the ontological group representing the genes responsible for “cellular response to hormone stimulus”. *ESR1* (estrogen receptor 1), responsible for the expression of proper estrogen receptors encodes a DNA binding protein activating the transcription process. After estrogen is bound to the ESR1 receptor, the metabolic pathway responsible for ovarian follicle maturation begins. The highest level of *ESR1* expression was observed in the fetal period in the nuclei of growing oocytes (Chen et al. 2009). Lower *ESR1* gene expression in mature oocytes confirms its decline during the maturation process and highest level at early stages of folliculogenesis. Other data suggest that reduction of ESR1 may be correlated with oocyte size and may lead to changes in the activity of the PI3K/AKT pathway (Artini et al. 2017). The *ESR1* gene is co-expressed with the next two identified genes: *AR* (androgen receptor) and *EGR2* (early growth response 2). *AR* carries information about a two-domain transcriptional activator protein (composed of androgen receptor and DNA binding domain). Androgen receptors play a key role in primary ovarian follicles (Ma et al. 2017). The action of androgens on early stages of folliculogenesis and their simultaneous expression with *IGF1R, IGF2*, and *IGFBP3* appears to be necessary for the early primordial ovarian follicle activation. *EGR2* encodes a transcription factor consisting of the three tandem C2H2-type zinc fingers. EGR gene family is responsible for the differentiation processes, managing growth and cellular functions. Decreased expression of the *EGR2* gene in mature oocytes confirms the results presented by Celichowski et al. in maturing oocytes, *EGR2* shows a notably stronger inhibitory effect on gene expression as compared to *EGR1* (Celichowski et al. 2018).

Tachykinin receptor 3 (*TACR3*) carries information about the neurokinin B receptor. TACR3 is a G protein-coupled receptor, mainly expressed by Leydig cells in the testis (Topaloglu et al. 2009). Inhibition of *TACR3* expression in the early ovary by Wnt-4 can promote female sex determination (Vainio et al. 1999). Mutations associated with loss of *TAC3/TACR3* gene functions cause IHH (isolated hypogonadotropic hypogonadism), characterized by a lack of sexual maturation and low circulating levels of LH and gonadal steroids (Young et al. 2010). Cyclin D2 (*CCND2*) is responsible for ovarian cell proliferation, most notably regulating the G1/S phase transition during the cell cycle. The highest level of its transcripts was observed in ovarian and testicle tumor cells (Tan et al. 2005). In embryonic development, a high rate of *CCND2* expression and proliferation is observed during early cortex and ovarian cord formation. Higher levels of *CCND2* expression were also observed at the beginning of pregnancy (Hummitzsch et al. 2019). During folliculogenesis, *CCND2* plays a key role in the proliferation of cumulus oophorus cells. Null mutation and block of *CCND2* expression in mice undermines the proliferation of cumulus cells, resulting in the growth of small follicles that are unable to ovulate (Sicinski et al. 1996). Increased expression of *CCND2*, as well as CTNND1 (catenin delta 1), is inhibited by LH through the activation of CD44 (van Montfoort et al. 2008). So far, there is no clear information about the cause of this gene’s expression in oocytes. The presence of CCND2 mRNA transcripts may be the result of their transport inside the oocyte in the process of two-way communication with the surrounding cumulus cells. The last of ten genes that were identified as differentially expressed is *TGFBR3* (transforming growth factor beta receptor 3). *TGFBR3* may be as a mediator of cancer progression and epithelial to mesenchymal transition factor. Beta-glycan encoded by this gene mediates signal transduction by members of the TGFβ (transforming growth factor—β) superfamily. In the group of CCs subjected to temporary hypoxia, increased expression of this gene was observed (Al-Edani et al. 2014). The decrease in *TGFBR3* expression in oocytes after IVM may be caused by the fact that the oocyte itself is not involved in the formation of corpus luteum blood vessels, for which the *TGFBR3* is largely responsible (Chermuła et al. 2018; Sarraj et al. 2007).

Lipid droplets in oocytes are important organelles for embryonal development of porcine embryos. Previously it was reported that removal of cytoplasmic lipid droplets (delipidation) in porcine embryos decrease the extent of injury during cryopreservation and enhance tolerance of porcine oocytes to chilling (Nagashima et al. 1994). However, delipidation itself could compromise embryo viability, as intracellular lipids are a source of oocyte energy and exist as complexes of ‘‘smooth endoplasmaic reticulum–lipid globules–mitochondria’’ in cells (Sathananthan 1994). Lipid droplets are the main source of energy for embryonic development (Sturmey and Leese 2003), and their number and distribution patterns in oocytes and embryos have been reported linked to the developmental competence of early embryos through the blastocyst stage (Yoneda et al. 2004). It was presented that delipation of oocytes had no effect on the cleavage ability of the partnenogenetic porcine embryos during the early developmental stages (2-cell and 4-cell stages), but the ability to form a blastocyst was significantly reduced, perhaps, because of the reduction in available lipid droplets and mitochondria or both (Niu et al. 2015). Lipid composition deposited in the oocyte has been shown to be accumulated during follicular development (Fair et al. 1997). Hyttel et al. (1997) observed that at the GVBD stage lipid droplets within the oocyte cytoplasm are increased in number and size. The total area of lipid droplets was similar between GV stage and in vitro matured oocytes, contrary to a number of lipid droplets. The number of lipid droplets decreased during the process of maturation, with their surface area progressively increasing. Niimura et al. (2002) consider that the increase in the number of lipid droplets in porcine oocytes is closely related to the resumption of meiotic maturation, regardless of in vivo or in vitro maturation. Our findings indicate that during in vitro maturation oocyte metabolized primary small lipid droplets. The total lipid content may have a potential role as reserve fuel and have decreases over the course of oocyte maturation in vitro.

Overall, this study paid particular attention to the comparison of expression of genes directly or indirectly involved in hormonal regulation of oocytes, as well as protein signaling pathways. After IVM, downregulation of expression was observed in all of the 25 analyzed genes belonging to two ontological groups: cellular response to hormone stimulus (GO:0032870) and cellular response to unfolded protein (GO:0034620). Next, 10 genes were identified as being the most altered in expression after the IVM process, with a possibility of playing key roles during COCs maturation. While maturation of oocytes in in vitro culture only allows for an approximate way to determine the levels of transcripts similar to those observed in in vivo conditions, it is still the best way to predict their developmental potential. The sensitivity of oocytes to changing the hormonal environment, as well as the basic pathways of cell signaling are both necessary for processes enabling the oocyte to be fertilized. The environment of oocyte growth is one of the most important factors in its development and maturation. The results may contribute to the broadening of knowledge about the processes associated with in vitro growth and maturation of oocytes.

Our research enabled identification and detailed genes expression analysis, which regulate very important for oocyte maturation cellular response to hormone stimulus and unfolded protein processes. The use of IVM in porcine oocytes can significantly contribute to the deepening of our knowledge about mechanisms regulating oocyte maturation resumption. Our research results in an animal model can significantly contribute to increasing the efficiency of in vitro maturation of human oocytes. IVM gives the possibility of increased ART safety in patients with PCOS. Another advantages of this method over controlled ovarian stimulation (COS) include lower drug costs and reduced treatment time.

Additionally, it also presents results of lipid droplet distribution and size analysis between immature and mature oocytes, potentially describing an interesting, non-transcriptomic approach of oocyte maturity determination. However, it needs to be noted that this manuscript presents an entry-level study, which, considering the high complexity of oocyte maturation, fertilization and early embryonic development processes, will certainly require further proteomic analysis to fully confirm the results and understand the processes that underlie the observed changes. The results of this study aim to serve as a basic molecular reference for further analyses that will be performed in the further stages of the project, potentially ultimately leading to improvement of understanding of the processes associated with important steps of assisted reproduction techniques.
